# Knowledge of Mental Illness and Its Associations Among Caregivers of Patients With Schizophrenia

**DOI:** 10.7759/cureus.66448

**Published:** 2024-08-08

**Authors:** Sanimar S Kochhar, Rakesh K Chadda, Mamta Sood, Rachna Bhargava

**Affiliations:** 1 Clinical Psychology, All India Institute of Medical Sciences, New Delhi, New Delhi, IND; 2 Psychiatry, Amrita Hospital, Faridabad, IND; 3 Psychiatry, All India Institute of Medical Sciences, New Delhi, New Delhi, IND

**Keywords:** side-effects, associations, diagnosis, medication, psychosocial, mental health literacy, knowledge of illness, mental illness, caregivers, schizophrenia

## Abstract

Background: Schizophrenia is a severe mental illness that greatly impacts the real-world functioning of patients. In India, caregivers are primarily responsible for their patients and function as their support system, often taking treatment decisions on their behalf. However, they may have insufficient knowledge of the illness, which can have a negative impact on their roles as effective caregivers. The knowledge of schizophrenia and its associations among caregivers has received very little research attention.

Aim: To examine the knowledge of mental illness and its socio-demographic and psychosocial associations among caregivers of patients with schizophrenia. The objectives were to assess the knowledge of mental illness and its treatment in caregivers of patients with schizophrenia, to assess the socio-demographic and clinical associations of this knowledge, and to assess the caregivers’ psychosocial variables associated with this knowledge.

Methodology: This cross-sectional observational data was taken from a larger study carried out between August 2018 and January 2021 at an urban tertiary care hospital in the capital city of India. One hundred fifty-eight patients with schizophrenia and their caregivers (n=158) were taken using purposive sampling. Knowledge of Mental Illness Scale was used to evaluate the knowledge and understanding of the illness and its treatment among caregivers. Caregivers coming to this institution in New Delhi were also assessed with respect to their demographic variables, caregiving experience, family functioning, coping strategies, available social support, psychological distress, quality of life, and spiritual, religious, and personal beliefs. The assessment also included demographic and clinical variables of the patients.

Results: Caregivers possessed relatively greater knowledge regarding the medication being taken (52.5%), its side effects (38%), and the diagnosis (36.1%). However, their knowledge was poorer with respect to the meaning and implications of the diagnosis (21.5%) and the purpose of the medication (10.1%). In multivariate regression analysis of these five domains with socio-demographic, clinical, and psychosocial variables; the knowledge of the diagnosis was associated with a family history of psychiatric illness in a second-degree relative, the total duration of treatment, and stigma in caregivers. The meaning of the diagnosis was associated with the environmental domain of quality of life and positive symptoms of the patient. Knowledge of medication was associated with the number of patient hospitalizations, caregivers supervising medications, caregiver age, and the total duration of illness. Knowledge of the purpose of medication was associated with the total duration of illness and patient positive symptoms. Knowledge of medication side effects was associated with the roles domain of family functioning, positive caregiving experience, patient negative symptoms, and the acceptance/redefinition domain of coping.

Conclusion: A range of associations were examined in this study. Correct knowledge of schizophrenia is necessary to promote timely help-seeking, preventing a longer duration of untreated psychosis and a poor prognosis. In research, knowledge of illness is a construct with immense potential applicability. In patient care, this knowledge may help caregivers participate in treatment planning, improve patient functioning, and support their patients toward better functional outcomes.

## Introduction

Schizophrenia is a severe psychiatric disorder affecting the thoughts, feelings, and behavior of an individual, rendering them out of touch with reality [1]. The clinical picture includes an onset in young adulthood with positive symptoms such as persistent delusions and hallucinations, disorganized thinking and behavior, negative symptoms such as apathy and social withdrawal, and cognitive difficulties. Thus, these symptoms profoundly impact the personal, familial, social, educational, and occupational functioning of those affected [2]. Caregivers are instrumental in providing care, assistance with activities of livelihood, and various forms of emotional and practical support [3]. It has been observed that many caregivers do not possess adequate knowledge of illness and skills to deal with this complex disorder [4]. A study on caregiver health education needs found that caregivers require assistance in the recognition, comprehension, and management of specific and common symptoms of schizophrenia [5]. They also seek information about new and emerging treatments and information regarding causes, course of illness, symptoms, medications, and their side effects in schizophrenia [4].

Mental health literacy is the knowledge of mental disorders, which helps in recognizing, managing, and preventing them. This encompasses skills such as searching for information, comprehending the causes and risk factors, seeking professional assistance, and adopting attitudes that align with help-seeking behavior [6]. Thus, knowledge of schizophrenia helps caregivers be effective in their role [7]. It also increases timely help-seeking and successful treatment before the disorder becomes severe and chronic and improves the patients’ rehabilitation [8]. As a result, it ultimately reduces the global burden caused by the disease. 

The knowledge of schizophrenia is a construct with manifold applicability in schizophrenia research. As a correlate, it is associated with help-seeking and treatment adherence in patients [9]. It can lead to fewer relapses, fewer hospitalizations, better patient functioning, an improved quality of life, and greater satisfaction with health services [10]. It can also promote effective caregiver coping [11]. Despite this, there have been very few studies conducted in this area. There is a clear need for the community to receive appropriate information about schizophrenia, both in terms of understanding the illness itself and how caregivers perceive the information they receive from health professionals [12]. Medication awareness is connected to treatment adherence, and many times, patients are dependent on their caregivers to supervise medications [13]. Caregivers need to be aware of the treatment being administered and its effects, as patients may lack insight.

There are many lacunae in research on caregivers’ knowledge of schizophrenia. There are studies carried out on the opinions of the general public, but very few among caregivers of patients with schizophrenia. There is ample research literature on psychoeducational studies; however, there is a dearth of publications about caregivers’ knowledge of illness and treatment. To the best of the author’s knowledge, there is so far only one quantitative study that has examined the caregiver’s knowledge of schizophrenia, taking it as a main variable, with a range of other variables (psychological well-being, burden and coping) [11]. Therefore, in the present study, the aim was to assess the knowledge of mental illness (KMI) and its associations among caregivers of schizophrenia patients. Specifically, there were three objectives: To assess the knowledge of mental illness and its treatment among caregivers of schizophrenia patients; to assess the socio-demographics and patient clinical variables associated with this knowledge; and to assess caregivers’ psychosocial variables associated with this knowledge. We have taken the knowledge of illness to include the knowledge about diagnosis, its meaning, knowledge of medication, its purpose and its side effects, according to Kotze et al. [14]. 

## Materials and methods

Setting and participants

This study utilizes data from a larger observational study with a short follow-up [15] conducted in the Department of Psychiatry of a tertiary care hospital in New Delhi. It is a reputable institution in the capital city of India. Patients from all socio-economic classes come here, with many having schizophrenia availing of the services.

Sample

The sample was taken using purposive sampling and comprised 158 patients with schizophrenia and their caregivers (n=158) from the outpatient department of the Department of Psychiatry. Formula (1) was used to calculate the sample size calculation. Formula (2) shows the actual calculation of sample size. The sample size was calculated for a larger study [15], of which this study is a part. Doval et al. [16] was the reference study. Taking a conservative value of the correlation as approximately 0.25, with a power of 90%, and α (type 1 error) as 5%, the required sample size was 158.

Sample Size Calculation, $n = \frac{(Z _{ 1-\beta} + Z _{ 1-\alpha/2})^{2}}{(\frac{r^{2}}{1-r^{2}})}$, (1)

where n: sample size required, alpha: 0.05 (level of significance), β=1−power of the test, r: correlation coefficient, Z_(1−α/2)_=desired confidence level, Z_(1−β)_: standard normal variate at 90% power (1−β=1−0.10=0.90).

Actual Calculation of Sample Size, $n = \frac{(1.26 + 1.96)^{2}}{(\frac{0.25^{2}}{1-0.25^{2}})}$, n=157.5≅158, (2)

where 1−β=90% (power); Z_(1−β)_=1.26; r=0.25 (approximately); Z_(1−α/2)_=1.96. Calculation using N Master 2.0 (Biostatistics Resource and Training Center, Department of Biostatistics, Christian Medical College, Vellore, India).

Inclusion and exclusion criteria

Patients with a diagnosis of schizophrenia as per ICD-10 criteria for greater than one year, regardless of gender, and within the age range of 18-55 years were included. Caregivers residing with the patient for more than one year; holding primary responsibility for the patient and of providing treatment supervision (such as managing medication and accompanying patients to the hospital); demonstrating proficiency in Hindi (the local language), understanding instructions for assessment and providing informed consent were included. Patients with comorbidities, such as disabling medical or psychiatric illnesses, substance dependence (except tobacco), or organic brain conditions, were excluded. Caregivers with a diagnosis of a disabling medical or psychiatric illness, without regular contact with the patient, and not residing with the patient were excluded. Caregivers with a family member diagnosed with a chronic and disabling medical or psychiatric illness in the same home unit were also excluded.

Ethical considerations

Ethical approval for the present work was received from the Institute Ethics Committee, the All India Institute of Medical Sciences, New Delhi (Approval No.: IECPG-204/10.05.2018).

Procedure

The research was initiated after obtaining ethical approval from the AIIMS Institute Ethics Committee. Patients with a diagnosis of schizophrenia (determined by a consultant psychiatrist), visiting the OPD of psychiatry were approached and screened. If they were accompanied by a caregiver and met the inclusion criteria; the purpose of the study was explained to them. Patients and caregivers providing informed consent were recruited, and the scales were administered to them. The patients and caregivers completed the assessment in one sitting, with breaks in between. The average time taken for the assessment, including all scales, was three hours, out of which the assessment of the KMI questions took only five minutes. The socio-demographics of patients and caregivers and the clinical information of the patients were recorded. An assessment of patients’ positive and negative symptoms was also carried out. The primary measure, the Knowledge of Mental Illness Scale, was applied to the caregivers. In caregivers, the Experience of Caregiving Inventory was used to assess caregiving experiences, the Family Assessment Device (FAD) was used for assessing family functioning, the Coping Checklist was used for measuring coping, the Social Support Questionnaire-Hindi measured social support, the General Health Questionnaire-12 assessed caregiver distress, the World Health Organization Quality of Life (WHOQOL-BREF) measured quality of life, and the WHOQOL-SRPB measured spiritual, religious, and personal beliefs.

Measures

Semi-Structured Proforma

A semi-structured proforma was developed to enquire about patient and caregiver demographics and patient clinical variables. This included the age, gender, marital status, education, and occupation of the patient and caregiver, as well as the caregiver’s relationship with the patient. The family income, area of residence, and type of family were also asked. The patient’s age of illness onset, type of onset, total duration of illness, total duration of treatment, and number of hospital admissions, if any, for psychiatric illness were also enquired. It was also probed as to whether there was any family history of psychiatric illness and in whom. The time spent in caregiving per day (hours) and the duration of being in the caregiver’s role (years) was enquired, and it was also asked if the caregiver was supervising the medicines of the patient. The number of family members involved in the patient’s care was also asked.

The Knowledge of Mental Illness (KMI) Scale [14] was the main instrument utilized in this study. It covers facets like diagnosis, etiology, medication, and treatment of mental disorders. Its five items are rated with yes or no responses. The questions are asked in a semi-structured manner. The questions include knowing the diagnosis, knowing what it means, knowing what medication the patient is on, knowing what the medication is for, and knowing what the side effects of the medications are. Specifically, yes/no responses were obtained for this scale. This scale was translated into Hindi, the local language, for use. This is not a validated scale, and its psychometric properties are unknown. However, the procedures were followed as indicated in the reference article [14]. We carried out a semi-structured interview where we asked the five questions. The responses were corroborated against the clinical files and outpatient department cards of the patients, for instance, to see if they knew their diagnosis and medication. Based on this, we assigned yes/no responses. The responses given by the caregivers did increase our understanding of their knowledge of the illness. If the caregivers could name schizophrenia, it was scored a yes for the diagnosis. If the caregiver got the primary medication correct and could name one or two major side effects; these were scored as a yes for knowledge of medication and medication side effects; respectively. In correspondence with the original author of the scale, with respect to the meaning of the diagnosis, if the caregiver understood what the illness is; and could at least mention some of the symptoms, we accepted it and scored it as a yes. This was asked by probing for their understanding of the illness, what its characteristics are, and what symptoms/phenomena are seen in this illness. With respect to the purpose of the medication, if they understood the illness and then said that the medication was to help for those symptoms, we scored it as a yes. If they simply said to get well/because the doctor said so, but could not link the treatment to the symptoms or the psychiatric illness, it was marked no. For instance, a valid response could consist of the illness being due to a chemical imbalance in the brain and the medication serving to correct that imbalance. We were consistent with what we found acceptable, and we scored accordingly.

The Experience of Caregiving Inventory [17] is a measure assessing both positive and negative caregiving experiences, based on the stress-appraisal-coping framework. It has 66 items rated on a five-point Likert scale. It has eight negative and two positive domains in caregiving appraisal. The negative domains consist of difficult behaviors, negative symptoms, stigma, problems with services, effects on the family, need for backup, and dependency and loss. These can be summed up to give a total negative score and a total positive score, respectively. The internal consistency of the domains ranges from 0.74 to 0.91 for the actual inventory.

The Family Assessment Device (FAD) [18] is a self-report scale measuring family functioning on a four-point scale. It is based on the McMaster model of family functioning. It has 60 items and seven subscales: problem-solving, communication, roles, affective responsiveness, affective involvement, behavioral control, and general family functioning. Higher scores indicate worse family functioning. Internal consistency in the subscales ranges from 0.72 to 0.92 for the actual inventory.

The Coping Checklist [19] is a questionnaire with 70 items covering various coping strategies used to cope with stress. It comprises seven domains: problem-focused (problem-solving), emotion-focused (positive distraction, negative distraction, acceptance/redefinition, religion/faith, and denial/blame), and social support, which encompasses both problem-focused and emotion-focused components. The Cronbach’s alpha for the actual inventory is adequate (0.86 for the complete instrument), and its test-retest reliability for one month has been found to be 0.74.

The Social Support Questionnaire-Hindi [20] was utilized for measuring social support. It is adapted in Hindi from Pollack and Harris (1983) [21]. This is a four-point Likert scale with 18 items. Higher scores indicate higher social support. The modified scale has high test-retest reliability for the actual inventory (r=0.91, p<0.01).

The General Health Questionnaire-12 (Hindi Version) [22] was applied to measure caregiver psychological distress. It is a screening tool used in a variety of settings that can identify psychiatric cases. Each item is scored on a two-point scale. A cut-off score of ≥2 indicates probable psychiatric morbidity in the caregiver. For the Hindi translation of the 60-item full scale, the tool can differentiate the normal population from patients statistically; with p <0.01 showing good validity. Also, by the split-half method, the correlation was 0.78 for patients and 0.73 for the normal subjects, demonstrating the reliability of the measure.

The WHOQOL-BREF (Hindi Version) [23] was applied to measure caregivers’ quality of life. Its four domains are physical health, psychological health, social relationships, and environment. It has 26 items, which are scored from one to five, with a total score range of 26-130. It has good psychometric properties, similar to those of the complete version. The Cronbach alphas for the four domains range from 0.66 to 0.84. The discriminant validity is also comparable, with similar values and significant differences between ill and well participants. The test-retest reliabilities ranged from 0.66-0.87 for the different domains. 

The WHOQOL-SRPB (Hindi Version) [24] measures spiritual, religious, and personal beliefs. It has 32 items in eight domains: spiritual connection, meaning and purpose in life, experiences of awe and wonder, wholeness and integration, spiritual strength, inner peace, hope and optimism, and faith. It is a five-point Likert scale. The internal consistency was in the range of 0.77 to 0.95, and it was 0.93 for the complete actual inventory.

The 34-item Scale for the Assessment of Positive Symptoms (SAPS) was applied to measure positive symptoms in patients with schizophrenia [25]. Positive symptoms assessed include hallucinations, delusions, bizarre behavior, and positive formal thought disorders. The coefficient alpha for this scale has been reported to be 0.483.

The 25-item Scale for the Assessment of Negative Symptoms (SANS) was utilized to measure negative symptoms [26]. These include affective blunting, alogia, avolition or apathy, anhedonia or asociality and attentional disturbances. The coefficient alpha for this scale as a whole has been reported to be 0.855.

Statistical analysis

The licensed statistical software SPSS version 25.0 (IBM SPSS Statistics for Windows, Version 25, Armonk, New York, USA) was used to analyze the data. The normalcy of the data was assessed using the Kolmogorov-Smirnov test, and descriptive statistics were utilized to summarize the data. For continuous variables, the mean and standard deviation and median and inter-quartile range were computed for normally distributed and non-normally distributed data, respectively. For categorical data, frequency (N) and percentages are provided. 

Since the KMI is a nominal scale with five domains consisting of two categories each (yes/no), we have utilized tests accordingly for all analyses. The associations of each of the five domains with demographic, psychosocial, and clinical variables were examined using Chi-square or the Fisher Exact test for categorical variables. The explanatory variables have been put in the rows field, and the response variable (the respective knowledge domain) has been put in the columns field. Since the row variables are explanatory, row percentages have been used for interpretation. The comparisons of the categorical domains of knowledge with continuous and normal variables were carried out by the two independent samples t-test, where Levene’s test was used to check the homogeneity of variance. The Mann-Whitney test was used for the comparison of the KMI domains with continuous and non-normally distributed variables. Statistical significance was kept at p<0.05, and the Bonferroni correction was applied for multiple exploratory comparisons. Since there were 62 comparisons in total, the corrected p-value came out to be 0.0008. Due to the large number of comparisons and associations, only results significant at p<0.05 are displayed in the tables.

All variables found significant up to 0.1 in the bivariate analysis were considered in the stepwise multivariable logistic regression, with the probability of entry as 0.05 and the probability of removal as 0.1. To find independently associated variables with knowledge of diagnosis, knowledge of the meaning of diagnosis, knowledge of medication, knowledge of the purpose of medication, and knowledge of medication side effects; and their strength of association; unadjusted and adjusted odds ratios with a 95% confidence interval were calculated.

## Results

The results pertaining to socio-demographics have been tabulated and described in detail in another publication by the same authors [15]. One hundred fifty-eight patients and their caregivers (n=158) were included in the study. Most of the patients were young adults in the age range of 18-35 (67.7%), with a mean age of 32.7 years (SD 8.9). 55.7% were males and 44.3% were females. Most of the patients were never married (65.2%). There was a preponderance of Hindu patients and caregivers (86.1%). Most patients had studied up to at least 10th grade (91.8%), with one-third being graduates. Most of the patients were unemployed (57%).

The mean age of caregivers was 45.6 (SD 16.0) years. Most of them were above the age of 35 (67.7%). 52.5% of the caregivers were parents, and 27.2% were siblings. The percentage of males and females was 53.8% and 46.2%, respectively. Most of the caregivers were married (69.6%) and had at least 10th-grade education (95.6%). The caregivers belonged to varied occupational backgrounds, with more than one-third being either professionals, skilled workers, or businessmen (38.6%). The families predominantly had an urban residence (70.8%) and were mostly nuclear families (64.6%). The average duration of the caregiving role was 7.7 years (SD 5.7). In clinical variables, the average duration of illness was 9.5 years (SD 6.3), and the average duration of treatment was 7.9 years (SD 5.8). The SAPS score (assessing positive symptoms) was 19.8 (SD 16.9), and the SANS score (assessing negative symptoms) was 38.3 (SD 17.1).

It was observed that 63.9% of the caregivers did not have specific knowledge regarding the name of the illness, i.e., schizophrenia. Most of the caregivers were not aware of the meaning of the illness/diagnosis or the implication of the term schizophrenia (in terms of symptoms or causation) (78.5%). The knowledge of the primary medication that the patient was taking was known by more than half (52.5%) of the participants. The purpose of medication (what the medication is for) was not known by nine caregivers out of ten (89.9%). Only 38% of caregivers were aware of medication side effects (Table 1).

**Table 1:** Caregiver knowledge about schizophrenia. Data is shown as n (%). KMI: knowledge of mental illness.

Questions on the KMI scale	Frequency (n)	Percentage (%)
Knowledge of diagnosis		
No	101	63.9
Yes	57	36.1
Knowledge of the meaning of diagnosis		
No	124	78.5
Yes	34	21.5
Knowledge of medication being taken		
No	75	47.5
Yes	83	52.5
Knowledge of the purpose of medication		
No	142	89.9
Yes	16	10.1
Knowledge of medication side effects		
No	98	62.0
Yes	60	38.0

In domain 1, the knowledge of diagnosis, higher family income (χ^2^=18.493, p=0.001), and caregiver education levels (χ^2^=19.461, p=0.001) were associated with greater knowledge of the diagnosis (schizophrenia). Similarly, the greater number of hospitalizations of the patient (χ^2^=8.992, p=0.025) was also associated with an increase in caregiver knowledge of diagnosis. A family history of mental illness (χ^2^=15.734, p=0.001), especially of schizophrenia, was associated with a better knowledge of diagnosis. This was especially the case for those caregivers whose first- or second-degree relatives (χ^2^=20.489, p<0.001) (apart from the patient) were afflicted. This association was highly significant, even after the Bonferroni correction. Greater caregiver age (z=−2.479, p=0.013) was associated with increased knowledge of diagnosis. Also, a longer duration of illness (z=−4.074, p<0.001) and treatment (z=−4.750, p<0.001) were associated with an increase in the level of knowledge of the diagnosis. These latter two variables had highly significant associations with this knowledge even after the Bonferroni correction. The duration of the caregiving role (z=−2.508, p=0.012) was also associated with an increase in the knowledge of diagnosis. In the psychosocial variables, normal role functioning in the family (z=−2.055, p=0.040) was associated with greater knowledge of diagnosis, also greater stigma levels (z=−2.407, p=0.016) were associated with increased knowledge. Higher scores on the environmental domain of quality of life (z=−2.095, p=0.036) (a better living environment) were also associated with better knowledge of diagnosis (Table 2).

**Table 2:** Domain 1: Knowledge of diagnosis. #1 USD=83.36 INR. *Significant at p<0.05, **Significant at p<0.01, ***Significant at p<0.001 (p=0.0008) (after Bonferroni correction).

Knowledge of diagnosis						
Variable	No (N (%))	Yes (N (%))	Total (N (%))		Χ^2^	p-value
Patient family income #						
Nil-5000 Rs.	4 (80.0%)	1 (20%)	5 (100.0%)		18.493	0.001**
5001-10,000 Rs.	6 (66.7%)	3 (33.3%)	9 (100.0%)			
10,001-20,000 Rs.	27 (87.1%)	4 (12.9%)	31 (100.0%)			
20,001-50,000 Rs.	44 (67.7%)	21 (32.3%)	65 (100.0%)			
>50,000 Rs.	20 (41.7%)	28 (58.3%)	48 (100.0%)			
Total	101 (63.9%)	57 (36.1%)	158 (100.0%)			
Caregiver education						
Middle school	7 (100.0%)	0 (0.0%)	7 (100.0%)		19.461	0.001**
10th grade	16 (94.1%)	1 (5.9%)	17 (100.0%)			
12th grade	26 (74.3%)	9 (25.7%)	35 (100.0%)			
Graduate	40 (56.3%)	31 (43.7%)	71 (100.0%)			
PG and professional	12 (42.9%)	16 (57.1%)	28 (100.0%)			
Total	101 (63.9%)	57 (36.1%)	158 (100.0%)			
No. of hospitalizations						
0	72 (69.9%)	31 30.1%)	103 (100.0%)		8.992	0.025*
1	23 (62.2%)	14 (37.8%)	37 (100.0%)			
2	4 (40.0%)	6 (60.0%)	10 (100.0%)			
3 or >3	2 (25.0%)	6 (75.0%)	8 (100.0%)			
Total	101 (63.9%)	57 (36.1%)	158 (100.0%)			
Family H/o mental illness						
No illness	69 (75.8%)	22 (24.2%)	91 (100.0%)		15.734	0.001**
Schizophrenia	1 (25.0%)	3 (75.0%)	4 (100.0%)			
Bipolar affective disorder (BPAD)/major depressive disorder (MDD)/substance abuse	3 (30.0%)	7 (70.0%)	10 (100.0%)			
Any other	28 (52.8%)	25 (47.2%)	53 (100.0%)			
Total	101 (63.9%)	57 (36.1%)	158 (100.0%)			
Psychiatric illness in whom						
Nobody	69 (75.8%)	22 (24.2%)	91 (100%)		20.489	<0.001***
First degree relative	7 (41.2%)	10 (58.8%)	17 (100.0%)			
Second degree relative	14 (38.9%)	22 (61.1%)	36 (100.0%)			
Third degree relative	11 (78.6%)	3 (21.4%)	14 (100.0%)			
Total	101 (63.9%)	57 (36.1%)	158 (100.0%)			
Variable		Mean		SD	z	p-value
Caregiver age						
Knowledge of diagnosis						
No		43.20		15.60	-2.479	0.013*
Yes		49.72		15.86		
Total duration of illness						
Knowledge of diagnosis						
No		7.88		5.14	-4.074	<0.001***
Yes		12.23		7.15		
Total duration of treatment						
Knowledge of diagnosis						
No		6.10		4.27	-4.750	<0.001***
Yes		10.95		6.90		
Duration of the caregiving role						
Knowledge of diagnosis						
No		6.69		4.72	-2.508	0.012*
Yes		9.54		6.79		
Roles domain of family functioning						
Knowledge of diagnosis						
No		2.31		0.52	-2.055	0.040*
Yes		2.14		0.55		
Stigma domain of caregiving experience						
Knowledge of diagnosis						
No		1.19		0.82	-2.407	0.016*
Yes		1.54		0.89		
Environmental domain of quality of life						
Knowledge of diagnosis						
No		14.90		2.73	-2.095	0.036*
Yes		15.81		2.87		

In domain 2, the knowledge of the meaning of diagnosis, higher patient family income (χ^2^=12.230, p=0.010) and higher caregiver education (χ^2^=10.054, p=0.040) were associated with greater knowledge of the meaning of diagnosis. A difference in the type of illness onset (χ^2^=13.027, p=0.008) was also associated with an increase in the knowledge of the meaning of diagnosis; as a longer onset period was associated with an increase in knowledge; with the greatest knowledge in patients having insidious (slow) onset. Also, caregivers who were supervising the patient's medications (χ^2^=4.846, p=0.028) had greater knowledge of the meaning of the diagnosis. Patients who had difficult behaviors as per the caregivers (t=−2.000, p=0.047) and those with a longer total duration of treatment (z=−2.059, p=0.039) had caregivers with greater knowledge of the meaning of the diagnosis. Greater stigma of illness (z=−2.979, p=0.003) and a better living environment (z=−2.186, p=0.029) were also associated with greater knowledge of the meaning of diagnosis; so was a higher level of positive symptoms (z=−2.497, p=0.013) in the patient (Table 3).

**Table 3:** Domain 2: Knowledge of the meaning of diagnosis. #1 USD=83.36 INR. *Significant at p<0.05, **Significant at p<0.01.

Knowledge of the meaning of diagnosis					
Variable	No (N (%))	Yes (N (%)	Total (N (%))	Χ^2^	p-value
Patient family income #					
Nil-5000 Rs.	5 (100%)	0 (0.0%)	5 (100.0%)	12.230	0.010*
5001-10,000 Rs.	7 (77.8%)	2 (22.2%)	9 (100.0%)		
10,001-20,000 Rs.	29 (93.5%)	2 (6.5%)	31 (100.0%)		
20,001-50,000 Rs.	53 (81.5%)	12 (18.5%)	65 (100.0%)		
>50,000 Rs.	30 (62.5%)	18 (37.5%)	48 (100.0%)		
Total	124 (78.5%)	34 (21.5%)	158 (100.0%)		
Caregiver education					
Middle school	7 (100.0%)	0 (0.0%)	7 (100.0%)	10.054	0.040*
10th Grade	16 (94.1%)	1 (5.9%)	17 (100.0%)		
12th Grade	29 (82.9%)	6 (17.1%)	35 (100.0%)		
Graduate	55 (77.5%)	16 (22.5%)	71 (44.9%)		
PG & professional	17 (60.7%)	11 (39.3%)	28 (100.0%)		
Total	124 (78.5%)	34 (21.5%)	158 (100.0%)		
Type of illness onset					
Abrupt	21 (95.5%)	1 (4.5%)	22 (100.0%)	13.027	0.008**
Acute	13 (81.3%)	3 (18.8%)	16 (100.0%)		
Sub-acute	20 (87.0%)	3 (13.0%)	23 (100.0%)		
Insidious	58 (68.2%)	27 (31.8%)	85 (100.0%)		
Not known	12 (100.0%)	0 (0.0%)	12 (100.0%)		
Total	124 (78.5%)	34 (21.5%)	158 (100.0%)		
Supervising medications					
No	73 (73.0%)	27 (27.0%)	100 (100.0%)	4.846	0.028*
Yes	51 (87.9%)	7 (12.1%)	58 (100.0%)		
Total	124 (78.5%)	34 (21.5%)	158 (100.0%)		
Variable		Mean	SD	t/z	p-value
Difficult behavior domain of caregiving					
Knowledge of the meaning of diagnosis					
No		1.66	0.85	-2.000	0.047*
Yes		2.00	0.91		
Total duration of treatment					
Knowledge of the meaning of diagnosis					
No		7.39	5.53	-2.059	0.039*
Yes		9.53	6.63		
Stigma domain of caregiving experience					
Knowledge of the meaning of diagnosis					
No		1.21	0.83	-2.979	0.003**
Yes		1.71	0.86		
Environmental domain of quality of life					
Knowledge of the meaning of diagnosis					
No		14.98	2.82	-2.186	0.029*
Yes		16.15	2.61		
Scale for assessment of positive symptoms					
Knowledge of the meaning of diagnosis					
No		17.42	14.38	-2.497	0.013*
Yes		28.21	22.22		

In domain 3, the knowledge of medication being taken, a greater number of hospitalizations of the patient (χ^2^=9.791, p=0.018) was associated with an increase in the knowledge of medication. The caregivers who were supervising the medications of their patients (χ^2^=6.093, p=0.014) also had greater knowledge of the medication. Higher patient (z=−2.524, p=0.012) and caregiver age (z=−2.921, p=0.003) were both associated with greater knowledge of medication. Total duration of illness (z=−3.504, p<0.001) and treatment (z=−3.316, p=0.001); and a greater duration of caregiving role (z=−2.131, p=0.033) were also associated with greater knowledge in this domain. In psychosocial variables, greater maladaptive coping by denial or blame (z=−1.974, p=0.048) by caregivers was associated with lesser knowledge of medication (Table 4).

**Table 4:** Domain 3: Knowledge of medication. *Significant at p<0.05, **Significant at p<0.01, ***Significant at p<0.001 (p=0.0008) (after Bonferroni correction).

Knowledge of medication							
Variable	No (N (%))	Yes (N (%))			Total (N (%))	Χ^2^	p-value
Number of hospitalizations							
0	58 (56.3%)	45 (43.7%)			103 (100.0%)	9.791	0.018*
1	12 (32.4%)	25 (67.6%)			37 (100.0%)		
2	2 (20.0%)	8 (80.0%)			10 (100.0%)		
3 or >3	3 (37.5%)	5 (62.5%)			8 (100.0%)		
Total	75 (47.5%)	83 (52.5%)			158 (100.0%)		
Supervising medications							
Yes	40 (40.0%)	60 (60.0%)			100 (100.0%)	6.093	0.014*
No	35 (60.3%)	23 (39.7%)			58 (100.0%)		
Total	75 (47.5%)	83 (52.5%)			158 (100.0%)		
Variable			Mean	SD		z	p-value
Patient age							
Knowledge of medication							
No			30.64	7.72		−2.524	0.012*
Yes			34.53	9.42			
Caregiver age							
Knowledge of medication							
No			41.60	15.47		−2.921	0.003**
Yes			49.12	15.62			
Total duration of illness							
Knowledge of medication							
No			7.62	4.94		−3.504	<0.001***
Yes			11.10	6.90			
Total duration of treatment							
Knowledge of medication							
No			6.23	4.58		−3.316	0.001**
Yes			9.31	6.46			
Duration of the caregiving role							
Knowledge of medication							
No			6.62	4.85		−2.131	0.033*
Yes			8.71	6.24			
Denial/blame coping							
Knowledge of medication							
No			0.44	0.58		−1.974	0.048*
Yes			0.32	0.21			

In domain 4, the knowledge of the purpose of the medication, a higher number of patient hospitalizations (χ^2^=12.765, p=0.002) was associated with higher knowledge of the purpose of the medication. It was also seen that a greater number of family members caring for the patient (χ^2^=7.892, p=0.034) was associated with lesser knowledge about the purpose of medication. Greater duration of both the illness (z=−2.598, p=0.009) and its treatment (z=−2.216, p=0.027) were both associated with greater knowledge of the purpose of the medication, as were greater positive symptoms of the patient (z=−2.761, p=0.006) (Table 5).

**Table 5:** Domain 4: Knowledge of the purpose of medication. *Significant at p<0.05, **Significant at p<0.01.

Knowledge of the purpose of medication					
Variable	No (N (%))	Yes (N (%))	Total (N (%))	Χ^2 ^	p-value
Number of hospitalizations					
0	94 (91.3%)	9 (8.7%)	103 (100.0%)	12.765	0.002**
1	36 (97.3%)	1 (2.7%)	37 (100.0%)		
2	8 (80.0%)	2 (20.0%)	10 (100.0%)		
3 or >3	4 (50.0%)	4 (50.0%)	8 (100.0%)		
Total	142 (89.9%)	16 (10.1%)	158 (100.0%)		
No. of family members in care					
1	17 (81.0%)	4 (19.0%)	21 (100.0%)	7.892	0.034*
2	68 (94.4%)	4 (5.6%)	72 (100.0%)		
3	38 (82.6%)	8 (17.4%)	46 (100.0%)		
>3	19 (100.0%)	0 (0.0%)	19 (100.0%)		
Total	142 (89.9%)	16 (10.1%)	158 (100.0%)		
Variable		Mean	SD	z	p-value
Total duration of illness					
Knowledge of the purpose of medication					
No		8.86	5.61	−2.598	0.009**
Yes		14.69	9.16		
Total duration of treatment					
Knowledge of the purpose of medication					
No		7.34	5.16	−2.216	0.027*
Yes		12.38	9.05		
Scale for assessment of positive symptoms					
Knowledge of the purpose of medication					
No		18.27	15.94	−2.761	0.006**
Yes		32.75	19.98		

In domain 5, the knowledge of medication side effects, the female gender of the patient (χ^2^=4.485, p=0.034) was associated with greater knowledge of side effects in the caregivers; as was patient family income (χ^2^=12.756, p=0.009). In caregivers’ relationships with the patients, mothers, and spouses of the patients (χ^2^=12.696, p=0.026) had greater knowledge of side effects. Greater difficult behaviors in patients (t=3.479, p=0.001), as appraised by the caregivers, were associated with greater knowledge of medication side effects. Greater negative (t=3.334, p=0.001) and positive (t=2.275, p=0.024) caregiving experiences of the caregivers were also associated with lesser knowledge of side effects, as also higher negative symptoms (t=3.503, p=0.001) in the patients as assessed by the SANS. Higher caregiver age (z=−1.991, p=0.046) was also associated with greater knowledge in this domain. Moreover, impaired roles domain of family functioning (higher scores) (z=−3.073, p=0.002) showed a significant association with lesser knowledge of side effects. Caregivers of patients having greater negative symptoms as per the ECI (z=−1.980, p=0.048), caregivers having problems with services (z=−3.228, p=0.001), caregivers having greater effects on the family (z=−2.025, p=0.043) and greater need for backup (z=−3.069, p=0.002) were all associated with lesser knowledge of medication side effects; as were greater scores from caregivers in the patient dependency (z=−2.493, p=0.013) and loss (z=−2.328, p=0.020) domains of the experience of caregiving inventory. Caregivers utilizing adaptive coping strategies like acceptance and redefinition (z=−2.369, p=0.018) and caregivers having a better living environment (z=−3.397, p=0.001) had significantly greater knowledge of medication side effects (Table 6).

**Table 6:** Domain 5: Knowledge of medication side effects. *Significant at p<0.05, **Significant at p<0.01, SANS: Scale for the Assessment of Negative Symptoms.

Knowledge of side effects						
Variable	No (N (%))	Yes (N (%))		Total (N (%))	Χ^2^	p-value
Patient gender						
Male	61 (69.3%)	27 (30.7%)		88 (100.0%)	4.485	0.034*
Female	37 (52.9%)	33 (47.1%)		70 (100.0%)		
Total	98 (62.0%)	60 (38.0%)		158 (100.0%)		
Patient family income						
Nil-5000 Rs.	5 (100.0%)	0 (0.0%)		5 (100.0%)	12.756	0.009**
5001-10,000 Rs.	6 (66.7%)	3 (33.3%)		9 (100.0%)		
10,001-20,000 Rs.	25 (80.6%)	6 (19.4%)		31 (100.0%)		
20,001-50,000 Rs.	40 (61.5%)	25 (38.5%)		65 (100.0%)		
>50,000 Rs.	22 (45.8%)	26 (54.2%)		48 (100.0%)		
Total	98 (62.0%)	60 (38.0%)		158 (100.0%)		
Caregiver relationship						
Mother	17 (41.5%)	24 (58.5%)		41 (100.0%)	12.696	0.026*
Father	30 (71.4%)	12 (28.6%)		42 (100.0%)		
Brother	20 (71.4%)	8 (28.6%)		28 (100.0%)		
Sister	11 (73.3%)	4 (26.7%)		15 (100.0%)		
Spouse	12 (54.5%)	10 (45.5%)		22 (100.0%)		
Son/daughter/other	8 (80.0%)	2 (20.0%)		10 (100.0%)		
Total	98 (62.0%)	60 (38.0%)		158 (100.0%)		
Variable		Mean	SD		t/z	p-value
Difficult behavior domain of caregiving						
Knowledge of medication side effects						
No		1.92	0.86		3.479	0.001**
Yes		1.44	0.81			
Total negative score of caregiving						
Knowledge of medication side effects						
No		86.66	29.03		3.334	0.001*
Yes		70.20	31.83			
Total positive score of caregiving						
Knowledge of medication side effects						
No		29.02	7.91		2.275	0.024*
Yes		25.82	9.60			
SANS						
Knowledge of medication side effects						
No		41.86	16.56		3.503	0.001**
Yes		32.35	16.55			
Caregiver age						
Knowledge of medication side effects						
No		43.55	16.67		-1.991	0.046*
Yes		48.82	14.24			
Roles domain of family functioning						
Knowledge of medication side effects						
No		2.35	0.54		−3.073	0.002**
Yes		2.08	0.49			
Negative symptoms domain of caregiving						
Knowledge of medication side effects						
No		2.25	0.95		−1.980	0.048*
Yes		1.93	1.02			
Problems with services domain of caregiving						
Knowledge of medication side effects						
No		1.19	0.74		−3.228	0.001**
Yes		0.80	0.73			
Effects on the family domain of caregiving						
Knowledge of medication side effects						
No		1.20	0.77		−2.025	0.043*
Yes		0.95	0.78			
Need for backup domain of caregiving						
Knowledge of medication side effects						
No		1.57	0.67		−3.069	0.002**
Yes		1.18	0.73			
Dependency domain of caregiving						
Knowledge of medication side effects						
No		2.52	0.75		−2.493	0.013*
Yes		2.16	0.94			
Loss domain of caregiving						
Knowledge of medication side effects						
No		1.63	0.63		−2.328	0.020*
Yes		1.38	0.69			
Acceptance/redefinition domain of coping						
Knowledge of medication side effects						
No		0.61	0.16		−2.369	0.018*
Yes		0.65	0.16			
Environmental domain of quality of life						
Knowledge of medication side effects						
No		14.62	2.68		−3.397	0.001**
Yes		16.22	2.39			

After the Bonferroni correction, family history of mental illness (in whom) (domain 1), total duration of illness (domain 1), total duration of treatment (domain 1), and total duration of illness (domain 3) were the four variables that were highly significant at p=0.008.

In multivariate regression analysis, in domain 1 pertaining to knowledge of diagnosis, the family history of psychiatric illness in terms of kinship (OR: 4.69, 95% CI: 1.90-11.59, p=0.001), the total duration of treatment (OR: 1.19, 95% CI: 1.10-1.29, p<0.001) and the stigma domain of the experience of caregiving inventory (OR: 1.64, 95% CI: 1.06-2.54, p=0.027) were all independently associated with greater knowledge of diagnosis in caregivers. Further, we found that a family history of psychiatric illness in a second-degree relative had higher odds of having knowledge of the diagnosis as compared to no kinship with a history of psychiatric illness. In domain 2, regarding the meaning of the diagnosis, the environmental domain of quality of life (OR: 1.22, 95% CI: 1.04-1.43, p=0.015) and positive symptoms in the patient as assessed by the SAPS (OR: 1.03, 95% CI: 1.01-1.06, p=0.008) were both independently associated with greater odds of having the knowledge of the meaning of diagnosis in caregivers.

In domain 3, regarding knowledge of the medication being given, the number of patient hospitalizations and whether the caregiver was supervising the medication of the patient were both independently associated with knowledge of medication. Compared to no hospitalizations, caregivers of patients having one hospitalization (OR: 3.36, 95% CI: 1.40-8.04, p=0.007) had greater odds of having knowledge of the medication being given. Also, caregivers not supervising medications had lesser odds of having knowledge in this domain (OR: 0.40, 95% CI: 0.19-0.84, p=0.016). The duration of the caregiving role was not significantly associated with this domain after regression. However, both caregiver age (OR: 1.03, 95% CI: 1.01-1.06, p=0.015) and total duration of illness (OR: 1.19, 95% CI: 1.06-1.34, p=0.004) of the patient were found to be significantly associated with knowledge of medication.

In domain 4, regarding the knowledge of the purpose of the medication, the total duration of illness of the patient (OR: 1.12, 95% CI: 1.04-1.21, p=0.004) and patient positive symptoms (OR: 1.04, 95% CI: 1.01-1.07, p=0.008) were both independently associated with this knowledge. In domain 5, in terms of knowledge of medication side effects, the roles domain of family functioning was negatively associated with this knowledge (OR: 0.31, 95% CI: 0.15-0.64, p=0.001); as also the total positive score of caregiving (OR: 0.94, 95% CI: 0.90-0.98, p=0.008) and patient negative symptoms (OR: 0.96, 95% CI: 0.94-0.99, p=0.002) as assessed by the SANS. The acceptance/redefinition domain of coping was independently associated with greater odds of having knowledge of medication side effects (OR: 24.38, 95% CI: 2.10-283.22, p=0.011) (Table 7).

**Table 7:** Logistic regression analysis. SAPS: Scale for Assessment of Positive Symptoms; SANS: Scale for Assessment of Negative Symptoms; QoL: Quality of Life.

Variable	Unadjusted odds (95% CI)	p-value	Adjusted odds (95% CI)	p-value
Domain 1				
In whom				
0	1		1	
1	4.48 (1.52-13.17)	0.006**	2.16 (0.62-7.57)	0.229
2	4.93 (2.16-11.24)	0.000***	4.69 (1.90-11.59)	0.001**
3	0.86 (0.22-3.35)	0.822	0.71 (0.16-3.17)	0.649
Total duration of treatment	1.18 (1.10-1.26)	0.000***	1.19 (1.10-1.29)	<0.001***
Stigma domain of caregiving experience	1.63 (1.10-2.41)	0.015*	1.64 (1.06-2.54)	0.027*
Domain 2				
Environmental domain of QoL	1.17 (1.01-1.36)	0.033*	1.22 (1.04-1.43)	0.015*
SAPS	1.04 (1.01-1.06)	0.002**	1.03 (1.01-1.06)	0.008**
Domain 3				
No. of hospitalizations				
0	1		1	
1	2.69 (1.22-5.92)	0.014*	3.36 (1.40-8.04)	0.007**
2	5.16 (1.04-25.48)	0.044*	4.79 (0.85-26.94)	0.076
3 or >3	2.15 (0.49-9.47)	0.312	0.72 (0.12-4.47)	0.728
Supervising medications				
Yes	1			
No	0.44 (0.23-0.85)	0.014*	0.40 (0.19-0.84)	0.016*
Duration of the caregiving role	1.07 (1.01-1.14)	0.024*	0.89 (0.78-1.00)	0.058
Caregiver age	1.03 (1.01-1.05)	0.004**	1.03 (1.01-1.06)	0.015*
Total duration of illness	1.11 (1.04-1.18)	0.001**	1.19 (1.06-1.34)	0.004**
Domain 4				
Total duration of illness	1.13 (1.05-1.21)	0.001**	1.12 (1.04-1.21)	0.004**
SAPS	1.05 (1.02-1.08)	0.003**	1.04 (1.01-1.07)	0.008**
Domain 5				
Roles domain of family functioning	0.36 (0.18-0.69)	0.002**	0.31 (0.15-0.64)	0.001**
Total positive score of caregiving	0.96 (0.92-1.00)	0.026*	0.94 (0.90-0.98)	0.008**
SANS	0.96 (0.94-0.99)	0.001**	0.96 (0.94-0.99)	0.002**
Acceptance/redefinition domain of coping	6.77 (0.82-55.83)	0.076	24.38 (2.10-283.22)	0.011*

## Discussion

This study assessed the knowledge of mental illness and its treatment in caregivers of patients with schizophrenia. It also assessed the various associations of this knowledge. The Knowledge of Mental Illness Scale by Kotze et al. was used in this study [14]. Regarding the knowledge of the diagnosis or the name of the disorder, in our study, it was observed that 63.9% of the caregivers did not know the name of the disorder. In an early study by Wray, 63% of the caregivers had positive or well-informed scores on knowledge of the diagnosis; however, 47% of the respondents were not informed about the diagnosis [12]. In the stated study, most caregivers learned about the diagnosis from other sources. Many, in their study, understood the disorder as severe and leading to disability. Thus, there was considerable scope for improvement in this area. The low knowledge may be attributed to either the inadequacy of professionals in imparting this information, vague and incomplete advice, or compromised learning by the respondents, due to the use of jargon, language difficulties, and cultural differences [12,14]. Especially, in our largely Hindi-speaking setting, the term ‘schizophrenia’ may be difficult to retain in memory even if mentioned.

Our results concur with the studies of Anab et al. and Magaru et al., where 75% and 72% of caregivers, respectively, did not have any previous knowledge regarding schizophrenia and learned only through their experiences with patients [7,27]. Most caregivers in these studies also did not feel that sufficient education was imparted about the illness. These studies, conducted in Papua New Guinea and Kenya, revealed a lower level of knowledge of diagnosis compared to our own study, potentially due to cultural and developmental differences. However, our findings on knowledge of diagnosis differ significantly from vignette-based studies like Ediriweera et al., where schizophrenia was identified as a disorder by 72% of caregivers [28]. This discrepancy may be due to the different methodologies used. In a vignette, a mental disorder can be identified; however, understanding the correct diagnosis on a rating scale may require a different level of mental health literacy. However, in a study from China with a vignette-based methodology, only 28.5% of the caregivers correctly described schizophrenia [8]. The information regarding the disorder's name could have been better in our study as well. Results were also somewhat similar to other studies from developing nations like Blessing from Nigeria [29] and Ghourmulla and Khan from Saudi Arabia [30], with average and low levels of knowledge, respectively. The differences were in the nature of the samples (higher education in the former and females below 30 years in the latter).

In terms of the meaning of the diagnosis, it was considered reasonable for the caregivers to comprehend some valid symptoms of the disorder. In our study, more than three-quarters of the caregivers (78.5%) could not cite any meaning or implication of the word schizophrenia (in terms of symptoms or causation). Our results were similar to those of Wray, where 59% of the respondents could not correctly identify the salient symptoms of the disorder [12]. In the study by Magaru, the caregivers were able to describe the negative and positive symptoms well [27]. Many times, florid symptoms are salient in the memories of the caregivers. However, in the study by Anab et al., disorganization symptoms were mentioned most often [7]. In our study, the second highest number of incorrect answers or no responses concerned the meaning and interpretation of the diagnosis. Similarly, another study found that the category with the highest number of incorrect responses was symptoms [10]. Wang et al. [31] also found that there were less than 50% correct responses on the etiology and symptoms of schizophrenia, indicating misconceptions in these areas. 

In findings related to the knowledge of the medication being taken by the patients, in our sample, under half of the caregivers (47.5%) did not have knowledge about the primary medication being taken. Our findings are supported by the study by Wray, where this figure was 27%, and where inadequate information was provided as per the caregivers [12]. A recent Indian study by Tripathi et al. revealed that while most caregivers (>90%) were aware of the domains related to past medical records and re-visiting instructions, only around 50% were aware of the details of the prescribed medication [13]. In the hospital where that study originated, informing patients and caregivers about the medications was prioritized [13]. Similarly, in our setting, caregivers may have heard the name of the prescribed drug from the doctors. Correct knowledge of prescription medication is a prerequisite for avoiding errors in consumption and supervision. Knowledge of atypical drugs was less seen in Wang et al. [31].

Regarding the knowledge of what the medication is for and its purpose, our study found that a vast majority of the sample (89.9%) had no knowledge of it. This was the category with the maximum number of wrong responses. Simply for getting okay was not considered a valid response. In the study by Wray, the caregivers knew that leaving medication may impact the illness course badly [12]. Another study also found that some of the most incorrect responses related to knowledge of treatment [10]. In the study by Tripathi et al., awareness of the purpose of the current prescription was present, as the reason for the prescription of each medication given was told to the patient and the caregivers [13]. Therefore, 31.21% of the caregivers in that study knew what the medication was for. It is likely that this information needs to be made more explicit in other settings as well.

Regarding the knowledge of medication side effects, our study found that 62% of the caregivers did not correctly know the side effects of the main medication or medications given. Similarly, the respondents in Wray's study also lacked knowledge about long-term adverse effects and strategies for managing side effects [12]. In the Anab et al. study, the side effects mentioned most commonly were tremors (42.5%) [7]. In the Tripathi et al. study, only around 50% of the caregivers demonstrated knowledge in the domain of medication side effects [13]. In our study, 38% knew about the side effects, which they may have seen occurring. Overall, it was seen in our study that caregivers possessed significantly greater knowledge about the medications and their side effects compared to the meaning of the diagnosis and purpose of the medications. Since the choice of antipsychotic depends on the side effect profile, caregivers should be able to identify side effects in patients if required.

One of the objectives of the present work was also to study the associations of various socio-demographics, clinical variables, and psychosocial variables with the various domains of the knowledge of illness. In bivariate results, in domain 1, regarding the knowledge of diagnosis, higher family income and higher caregiver education were associated with greater knowledge. Both give greater access to resources and education improves critical thinking skills. Regarding caregiver education, contrary to our findings, Tripathi et al. found no clinically significant relationship, although there were weak positive correlations of knowledge with SES and caregiver education [13]. In another exploratory study, education was found to be a predictor of knowledge among siblings of patients with psychosis [32]. The results were also supported by Anab et al., who found a high positive correlation between caregiver education and knowledge [7]. Thus, the more educated the caregivers are, the better they comprehend the medical history of the patient in terms of cause, treatment, and prognosis. Education adds to their depth of knowledge. Most of the caregivers in our study were well-educated. Our findings are also supported by other literature, which shows a significant positive correlation between higher education and increased knowledge of illness among family caregivers [9-11]. In the latter two studies, caregiver knowledge was observed to be higher than that of other groups, like lay community members and caregivers of other medical conditions. Wang et al. also found that caregivers with at least a university education had greater knowledge of the disorder compared to those educated up to middle school or below [31].

A greater number of patient hospitalizations increases the patients’ and the caregivers’ experience with the illness; and possibly gives greater exposure to the term schizophrenia being mentioned many times during the ward stay of the patient. A family history of mental illness, especially schizophrenia, in first- or second-degree relatives of the patient is associated with greater knowledge of the illness; as there is informational sharing in the family and vicarious learning of the illness through the experiences of other afflicted relatives. This latter variable was highly associated with the knowledge of the diagnosis, i.e., schizophrenia. Another factor associated with greater knowledge was higher caregiver age, as with increasing age comes the accumulation of greater knowledge about a variety of issues, which may include knowledge of a loved one’s illness.

A longer duration of the illness and its treatment, understandably, is associated with greater knowledge of the correct diagnosis, as caregivers learn more and more about the illness from both chronic patient symptoms and from their interactions with mental health professionals. Both of these variables were highly significant. The findings were supported by Anab et al., who found a similar association with the duration of illness in patients [7]. Caregivers may also develop a good understanding from experience. The results differed from another study that found no significant association between the duration of illness and the knowledge of caregivers [9]. A longer duration of caregiving role may similarly increase the chances of knowing the correct diagnosis, i.e., schizophrenia.

In the psychosocial variables associated with domain 1, impaired role functioning in the family was associated with lesser knowledge of the diagnosis. With unhealthy family functioning in terms of lack of clear demarcation of each family member’s role and responsibility, possibly have a lesser chance of correctly knowing about and understanding the illness. Also, in terms of greater stigma levels, whether caregivers have faced stigma or have self-stigma, it is possible that they do some introspection and knowledge-seeking, which may increase their knowledge regarding schizophrenia. Conversely, the association may be due to greater knowledge leading to greater stigma once they realize the ramifications of this illness. Contrary to our study, Singh et al. [33] found that higher stigma was predicted by a lower knowledge of schizophrenia among caregivers; and Koschorke et al. [34] found no association between knowledge and stigma in caregivers. An association with the environmental domain of quality of life could be due to a better living environment contributing towards better education and access to resources, leading to better knowledge of the diagnosis. These findings were supported by Alghamdi and Khan, who found an indirect association between knowledge of illness and quality of life [30]. The findings regarding role functioning and the environmental domain of quality of life are novel findings that could be replicated in future research.

In domain 2, pertaining to the knowledge of the meaning of diagnosis, higher family income, caregiver education, total duration of treatment, stigma and the environmental domain of quality of life were all associated with a greater knowledge due to the reasons described earlier. Among the new findings in this domain, the type of illness onset, specifically, a longer onset period (i.e., insidious onset), was associated with greater knowledge of the meaning of diagnosis. A slow onset may help in the understanding of the illness and its meaning in terms of symptoms, and it may give a period of time for the caregivers to collect their resources and deal with the disorder. Also, caregivers who were supervising medications had greater knowledge of the meaning of the diagnosis, as they may have observed the relation between giving the medication and a corresponding decrease in the symptoms. In psychosocial variables related to knowledge of the meaning of diagnosis, it was found that more difficult behavior of the patient may also have helped caregivers to understand what this illness entails, i.e., the meaning of the diagnosis. Similarly, in clinical variables, greater positive symptoms of the patient, when exhibited, may increase caregivers’ understanding of what this illness consists of, improving their knowledge in this domain.

In bivariate results on domain 3, i.e., the name of the medication being taken, the number of hospitalizations may have increased this knowledge. The medical staff mentions the names of medications repeatedly during ward stays, possibly leading to greater knowledge in this regard. In the same manner, supervising patient medications every day can increase this knowledge in caregivers. Additionally, higher patient age, caregiver age, and the total duration of illness and treatment can similarly increase caregiver knowledge of medications, as these factors concomitantly increase caregiver knowledge in the long run; as can an increased duration of caregiving role. Another novel finding of this study was that maladaptive coping by using denial or blame was associated with lesser knowledge of medication. Since the caregivers were not accepting of the illness and its reality, many may not bother to find out the knowledge of the disorder as well as the medication being prescribed.

In domain 4, regarding the purpose of the medication, the number of hospitalizations of the patient and the experience of ward stays were again associated with an increase in the knowledge of the purpose of the medication. As explained earlier, the total duration of the illness and treatment also added to this knowledge, as the longer the patients have been taking their medication, the higher the chances of the caregivers knowing what the medication helps in. Also, greater positive symptoms add to this knowledge, as caregivers can see firsthand the reduction of these symptoms by taking the medication long-term. Generally, additional caregivers were associated with lesser knowledge of the medication’s purpose, possibly due to these family members having differing viewpoints regarding the care of the patient.

In domain 5, regarding the knowledge of medication side effects, as explained earlier, higher family income and caregiver age contributed towards greater knowledge of the illness in this domain; due to increased resources and increased understanding, respectively. Similarly, the environmental domain of quality of life may lead to better knowledge due to the reasons explained previously. Contrary to domain 2, in this domain, greater difficult behaviors by the patient were associated with lesser knowledge of medication side effects. A possible explanation could be that some of the so-called difficult behaviors of the patient may be due to side effects that the caregiver does not know about. Impaired role functioning in the family of the caregiver was associated with lesser knowledge of medication side effects. This can be possibly due to impaired allocation of responsibility in the family, resulting in no one knowing about the medication's side effects.

Caregivers of female gender patients had more knowledge of medication side effects, possibly due to female patients being more expressive about their issues with the medication. In terms of kinship, mothers and spouses of the patients were seen to have better knowledge of medication side effects. This may be due to the closer relationships of these two kinship types, resulting in patients confiding in them about their issues, such as problematic side effects. Due to this close relationship, mothers and spouses may also be the ones supervising the medications, resulting in greater knowledge of their side effects. Wang et al. also found that other relationships, like siblings, have a poorer understanding of the illness, as they are less close in terms of kinship [31]. Higher positive and negative caregiving experiences were associated with lesser knowledge of medication side effects. Although the latter association may be understandable, the former was an unexpected finding, which may be further explored in future research. Greater negative symptoms, as assessed by the SANS, were associated with lesser knowledge of medication side effects, possibly due to some side effects being confounded by patient negative symptoms. A high negative symptom score, as per the ECI, also found the same results. Caregivers experiencing problems with mental health services were seen to have lesser knowledge of medication side effects, possibly because the services have not informed them about these potentially occurring. Also, caregivers experiencing the effects of the illness on the family were seen to have lesser knowledge in this domain. Lack of this knowledge may also possibly be causing these effects on the family. Additionally, caregivers having a greater need for backup and support regarding the patient had lesser knowledge in this domain, probably because they did not know how to manage the patient and manage any side effects that may be occurring. Caregivers of patients with greater dependency needs had lesser knowledge in this regard. Not knowing the side effects can lead to further patient dependency on their caregivers. Caregivers having higher scores on the loss experienced due to the patient’s illness also had lesser knowledge of medication side effects. It is possible that they experience loss as they do not comprehend the patient's issues caused by not knowing the side effects fully. Caregivers displaying adaptive coping by acceptance/redefinition of the illness showed greater knowledge of medication side effects. It is possible that caregivers who accept and redefine the illness in positive terms are also diligent enough to figure out or learn about these side effects. There are no published studies in the literature to compare these findings with.

In the results of the regression analysis, in domain 1, pertaining to the knowledge of the diagnosis, second-degree relatives having a psychiatric illness were most likely to affect the knowledge of diagnosis among the caregivers, as it may be a part of learning obtained from family conversations. The total duration of treatment was also highly associated with knowing the diagnosis, possibly due to increased interactions with mental health professionals over the course of time. Greater stigma in caregivers was associated with increased caregiver knowledge, as caregivers may have introspected and searched for information due to their stigma experiences. In domain 2, regarding the meaning of diagnosis, the environmental domain of quality of life was associated with this variable, possibly due to better learning opportunities and access to resources. Greater positive symptoms in the patient, as assessed by the SAPS, may have helped the caregivers learn the meaning of the diagnosis through observation.

In domain 3, regarding the knowledge of the medication being given, it was seen that even a single experience with hospitalization led to greater knowledge of the medication, possibly due to ward stay experiences. Caregivers who were not supervising the patient’s medications had lesser knowledge of medications, due to a lack of direct personal experiences of giving the medicine to caregivers. On the contrary, higher caregiver age and greater total duration of illness were also associated with increased knowledge of medication due to the accumulation of personal experiences. In domain 4, pertaining to the knowledge of the purpose of the medication, the total duration of illness and patient positive symptoms as assessed by the SAPS may have helped the caregivers understand the purpose of the medication better.

In domain 5, pertaining to the knowledge of medication side effects, the impaired role domain of family functioning was associated with lesser knowledge in this regard; as was the total positive score of caregiving. It is likely that if the caregivers’ experience of caregiving was positive, they may not have enquired about side effects. Also, the patient's negative symptoms assessed by the SANS were associated with lesser knowledge of this domain, as negative symptoms may overlap with the side effects, leading to lesser knowledge about them. On the contrary, coping by acceptance and redefinition was highly associated with better knowledge of medication side effects, as caregivers coping in a positive manner with the illness may have studied or learned about these effects by searching or by asking others or mental health professionals. 

This study has several important implications. The knowledge of diagnosis and its meaning were found to be inadequate in this study. Throughout the treatment process, healthcare providers should emphasize the name of the illness, so that the patients and the caregivers are not at a loss regarding the diagnosis. They should also be encouraged to learn more by searching for information from reliable sources. Audio-visual and self-instructional materials about the illness could be provided to caregivers to reinforce their learning. Regarding the medications and what they are for, the prescribing doctor should ensure that the names of the medications prescribed and their purpose are clearly delineated. The correct manner of supervising the medications, their probable side effects and how to manage them should also be informed. As seen from the results of the regression analysis, stigma and the knowledge of diagnosis were associated. Policymakers and community leaders should work towards addressing the stigma faced by caregivers, such that caregivers are armed with correct knowledge of the illness so that they can also sensitize others. These stigma reduction measures should continue long-term. By clearly demarcating each family member’s role and responsibility, patient management and knowledge of illness can be improved. Lastly, enhancing adaptive coping in caregivers, such as by acceptance and redefinition of the illness in positive terms, can lead to information seeking and better knowledge in caregivers.

Future research could focus on working to observe the dynamic interaction of all variables. For instance, a long follow-up could be carried out to document changes in caregiver knowledge. Paying attention to the cultural background of caregivers may also be useful. A greater number of studies need to be conducted in this area. The knowledge of schizophrenia is a potential target for assessment, and many more research questions on the knowledge of mental illness can be constructed.

This study was exploratory in nature, focusing on the knowledge of mental illness and providing information on caregivers' level of knowledge about schizophrenia. The strengths of this work include a fair sample size, exploring an area that is infrequently studied, and several relevant associations. The patient’s diagnosis was checked against clinical files, and socio-demographic and clinical data were collected to complement the findings. The findings are expected to shed light on the areas of awareness and unawareness in caregivers’ knowledge of schizophrenia. The focus was on schizophrenia per se, not general mental illness. The unique cultural setting adds value to the work. 

All patient clinical variables taken were significant in the bivariate analysis (number of hospitalizations, family history of mental illness (in whom), total duration of illness and treatment, supervising medications, type of illness onset, number of family members in care, SAPS and SANS scores). However, the type of onset and number of family members in care were insignificant after multivariate analysis.

There were some limitations in this study, mainly that the Knowledge of Mental Illness Scale is not a validated scale and its psychometric properties are unknown. However, it was brief, easy to administer, and easy to comprehend. Also, we do not know how much knowledge caregivers had before or from where they got this knowledge. However, it is quite likely that they may be having some prior knowledge, as most of these caregivers have been dealing with the illness of their patients for many years, as this illness is often chronic. These are intercurrent factors that were not controlled for; and form a limitation of this study. The cross-sectional data does not provide evidence of causation. The sample was from a single location (the OPD of a tertiary care institution) and was purposive; hence, generalization needs to be done with caution. Purposive sampling may be a reason for selection bias. Caregivers included in this study tended to be more educated, with higher family incomes, urban residence and nuclear families, as compared to the rest of the country; as they were coming to a tertiary care institution in the capital city of India. We have tried to correct for this bias to some extent by striving to include a broader spectrum of caregivers who did not fall into these categories. However, this bias could not be completely eliminated. This could result in reduced generalizability and external validity of the study. Also, these caregivers were in contact with mental health services and knowledge may differ for those not availing these services. Thus, selection bias was one of the limitations of this study. Other biases operational in this research could include recall bias, interview bias, non-response bias, ascertainment bias, and social desirability bias. For recall bias, the information was corroborated with preexisting medical records. We also tried to probe for information to aid recall. For interview bias, first, trust was established and the questions were asked in the same manner and were neutral and non-judgmental. They were not steering in any direction. A single interviewer asked all the questions and assigned the ratings to avoid interviewer bias. For non-response bias, we gave reminders and appointments were given at the caregivers’ convenience. For ascertainment bias, we had clear and predefined inclusion and exclusion criteria. The social desirability bias may also be operational here, as caregivers may have responded according to what they thought the researcher wanted to hear. This was addressed by assuring the participants of anonymity and confidentiality. Also, they were interviewed in a private room to ensure that they could express themselves freely. This study has a limited scope, as it is a preliminary effort in this area. 

## Conclusions

The current study evaluated the level of knowledge of illness among caregivers of patients with schizophrenia and focused on their understanding of the illness and its treatment. It also assessed the socio-demographic, clinical, and psychosocial associations of this knowledge. It found that caregivers possessed relatively greater knowledge in terms of the medication being taken and the side effects of the medications. Their knowledge was poorer regarding the meaning and implications of the diagnosis and the purpose of the medication. This study provides findings on the associations between caregivers' knowledge of illness and a range of other variables, which may help in designing future psychosocial interventions targeted at caregivers. As adequate knowledge of illness is a prerequisite for identifying early warning signs of relapse, ensuring compliance and providing correct supervision of medicines, healthcare providers should provide caregivers with comprehensive psychoeducation in this regard. Mental health professionals should meet caregivers’ expectations regarding information and provide a rationale for all treatment decisions. In the clinical environment, for increasing mental health literacy, healthcare providers could carry out frequent assessments in the form of pre-post tests, and self-help materials could be helpful. Here, healthcare providers should encourage biopsychosocial causation for mental illness, to help in aligning caregivers’ perspectives with those of the doctors, thereby fostering stronger therapeutic alliances. Policymakers and hospital administrators should implement health education programs and have regular audits for evaluating their efficacy. Policymakers and community leaders can also rope in media involvement for positive messaging and dispelling myths. Community leaders can also act through public education campaigns aimed at increasing awareness and reducing stigma. Policy development can include the provision that any caregiver who has not been imparted adequate psychoeducation can make a request to the hospital/mental health institution for the same. Mental illness is a complex interplay of biopsychosocial factors. It is the responsibility of the treating team to inform all concerned about the diagnosis and treatment in a clear and comprehensible manner. Greater knowledge of illness may lead to greater caregiver responsibility, better patient support; and increased hope for the future for both patients and their caregivers.
